# Supplementary material for: RNA sequencing analysis of FGF2-responsive transcriptome in skin fibroblasts
Source: PeerJ. 2021 Jan 15;9:e10671. doi: 10.7717/peerj.10671 (PMC7812929; doi:10.7717/peerj.10671)
Supplement: Supplemental Information 1 [file peerj-09-10671-s001.doc]

Table 1 Epidemiological data of healthy skin tissues

| Subject | Age | Sex | Region |
| --- | --- | --- | --- |
| 1 | 42 | F | Face |
| 2 | 39 | M | Face |
| 3 | 36 | F | Breast |
| 4 | 35 | M | Breast |
| 5 | 41 | F | Face |

Table 2 Sequences of primers for real-time PCR

| Gene | Forward | Reverse |
| --- | --- | --- |
| TGF-β 1 | 5’-GGCTTTCGCCTTAGCGCCCA-3’ | 5’-CTCGGCGGCCGGTAGTGAAC-3’ |
| Smad2 | 5’-GCAGAATACCGAAGGCAGACT-3’ | 5’-TTGAGCAACGCACTGAAGG-3’ |
| Smad3 | 5’-GAGAAACCAGTGACCACCAGATG-3’ | 5’-TAGGAGATGGAGCACCAGAAGG -3’ |
| Notch1 | 5’-GCTACGAGTGTGCCTGTGA-3’ | 5’-AGCCATTGATGCCCTCCTC-3’ |
| Jagged1 | 5’-GATGTCACCAGGTCTTACTAC -3’ | 5’-GTATATCTTCAGCAGAAATGG-3’ |
| Jagged2 | 5’-CACTGCTCCTGGCTGTCAC-3’ | 5’-AGGCACCACACAGCACAG-3’ |
| Delta-1 | 5’-GGTGGAGAAGCATCTGAA-3’ | 5’-CTTCCATTTTACACCTCAGTT G-3’ |
| COL1A1 | 5’-CATCTGGTGGTGAGACTTGC-3’ | 5’-TCCTGGTTTCTCCTTTGG-3’ |
| COL3A1 | 5’-GTCCCAGCGGTTCTCCA-3’ | 5’-CCCCGTGCTCCAGTGAT-3’ |
| CTGF | 5’-GTTTGGCCCAGACCCAACTA-3’ | 5’-GGCTCTGCTTCTCTAGCCCTG-3’ |
| Fibronectin | 5’-ACAAGCATGTCTCTCTGCCA-3’ | 5’-TCAGGAAACTCCCAGGGTGA-3’ |
| α-SMA | 5’-CTGTTCCAGCCATCCTTCAT-3’ | 5’-CCGTGATCTCCTTCTGCATT-3’ |
| GAPDH | 5’GGAGCGAGATCCCTCCAAAAT-3’ | 5’-GGCTGTTGTCATACTTCTCATCG-3’ |
