# Supplementary material for: RNA sequencing analysis of FGF2-responsive transcriptome in skin fibroblasts
Source: PeerJ. 2021 Jan 15;9:e10671. doi: 10.7717/peerj.10671 (PMC7812929; doi:10.7717/peerj.10671)
Supplement: Supplemental Information 2 [file peerj-09-10671-s002.doc]

Table 1 Sequences of primers for real-time PCR

| Gene | Forward | Reverse |
| --- | --- | --- |
| ITGA10 | 5’- AACATCACCCACGCCTATTCC-3’ | 5’- GTTGGTAGTCACCTAAGTGGC-3’ |
| COL4A1 | 5’- GGGATGCTGTTGAAAGGTGAA-3’ | 5’- GGTGGTCCGGTAAATCCTGG-3’ |
| COL3A1 | 5’- GGAGCTGGCTACTTCTCGC-3’ | 5’- GGGAACATCCTCCTTCAACAG-3’ |
| LOX | 5’- CGGCGGAGGAAAACTGTCT-3’ | 5’- TCGGCTGGGTAAGAAATCTGA-3’ |
| PDGFA | 5’- CCAGGACGGTCATTTACG-3’ | 5’- GCAGCGTTTCACCTCCAC-3’ |
| TGFBI | 5’- CACTCTCAAACCTTTACGAGACC-3’ | 5’- CGTTGCTAGGGGCGAAGATG-3’ |
| GAPDH | 5’ GGAGCGAGATCCCTCCAAAAT-3’ | 5’- GGCTGTTGTCATACTTCTCATGG-3’ |
